# Transgenic rat models for mutagenesis and carcinogenesis

**DOI:** 10.1186/s41021-016-0072-6

**Published:** 2017-02-01

**Authors:** Takehiko Nohmi, Kenichi Masumura, Naomi Toyoda-Hokaiwado

**Affiliations:** 1 Division of Genetics and Mutagenesis, National Institute of Health Sciences, 1-18-1 Kamiyoga, Setagaya-ku, Tokyo, 158-8501 Japan; 2 Present address: Biological Safety Research Center, National Institute of Health Sciences, 1-18-1 Kamiyoga, Setagaya-ku, Tokyo, 158-8501 Japan

**Keywords:** Genotoxicity, Carcinogenicity, DNA damage, Organ specificity, *gpt* delta, *lacI*, Genotoxic carcinogens, Non-genotoxic carcinogens, Chemoprevention, Threshold

## Abstract

Rats are a standard experimental animal for cancer bioassay and toxicological research for chemicals. Although the genetic analyses were behind mice, rats have been more frequently used for toxicological research than mice. This is partly because they live longer than mice and induce a wider variety of tumors, which are morphologically similar to those in humans. The body mass is larger than mice, which enables to take samples from organs for studies on pharmacokinetics or toxicokinetics. In addition, there are a number of chemicals that exhibit marked species differences in the carcinogenicity. These compounds are carcinogenic in rats but not in mice. Such examples are aflatoxin B_1_ and tamoxifen, both are carcinogenic to humans. Therefore, negative mutagenic/carcinogenic responses in mice do not guarantee that the chemical is not mutagenic/carcinogenic to rats or perhaps to humans. To facilitate research on in vivo mutagenesis and carcinogenesis, several transgenic rat models have been established. In general, the transgenic rats for mutagenesis are treated with chemicals longer than transgenic mice for more exact examination of the relationship between mutagenesis and carcinogenesis. Transgenic rat models for carcinogenesis are engineered mostly to understand mechanisms underlying chemical carcinogenesis. Here, we review papers dealing with the transgenic rat models for mutagenesis and carcinogenesis, and discuss the future perspective.

## Background

In modern industrial society, humans are inevitably exposed to a variety of chemicals. These chemicals are mostly important to sustain the society and improve the quality of life. Antibiotics and other pharmaceuticals are such examples and they significantly prolong longevity and improve peoples’ health conditions. However, there are a number of chemicals that might have adverse effects on humans. Such examples are cigarette smoke, air pollutants and contaminants in water and food. These adverse chemicals are sometimes linked to human cancer. Therefore, international organizations such as Organization for Economic Co-operation and Development (OECD) or World Health Organization (WHO) set up guidelines to evaluate the genotoxic and carcinogenic risk of chemicals [1]. Genotoxicity is regarded as an important biomarker for carcinogenesis because many human carcinogens are reactive to DNA and induce mutations in the target organs of carcinogenesis [2]. In mechanisms, mutations of many oncogenes and suppressor oncogenes are deeply involved in a variety of human cancer [3]. In general, it is believed that DNA reactive carcinogens impose cancer risk on humans even at very low doses [4]. Therefore, regulatory agencies in many countries pay strong attention to identify DNA reactive genotoxic agents to reduce the cancer risk related to exposure to environmental chemicals.

In 1970’s and 1980’s, genotoxicity of chemicals was examined mainly by in vitro short-term assays with bacteria and cultured mammalian cells. Although bacterial mutation assays, i.e., Ames test, is still the gold standard to identify DNA reactive genotoxic chemicals, in vitro genotoxicity assays have some limitations. Bacteria and most of cultured mammalian cells do not possess enough metabolic capacity to activate or inactivate chemical carcinogens [5]. So, rat liver homogenate, i.e., S9, is adopted to mimic the mammalian metabolism. However, some chemical carcinogens such as urethane give negative results in Ames test because of the inefficiency of S9 to activate the chemicals to ultimate mutagens [6]. On the other hand, non-carcinogenic chemicals such as 2,6-diaminotoluene (2,6-DAT) give positive results in Ames test probably because S9 does not have enough detoxication capacities [7, 8]. Recent survey revealed that in vitro mammalian genotoxicity assays such as chromosome aberration assays, gene mutation assays and micronucleus assays give many false positives, i.e., positives in the assays but negatives in rodent cancer bioassays [9]. Thus, in vivo genotoxicity is regarded more important than in vitro results in terms of decision making whether the particular chemical is genotoxic and carcinogenic to humans or not.

Classical in vivo genotoxicity assays are, however, very time consuming and target organs for the assays are quite limited. For example, “mouse spot test” uses developing embryo and detects mutations in the genes controlling the pigmentation of coat color of mice [10]. This test has been adopted into OECD Guidelines for the Testing of Chemicals as Test No. 484. If mutations are induced in the genes that control the pigmentation of coat color, the offspring will have spots of changed color in the coat. The frequency of such spots in the treated mice is compared to that of the spots in untreated mice. Although this assay certainly detects mutations in mice in vivo, the target organ for mutagenesis is only melanoblasts in embryo. Because very few people conduct the assays nowadays, it has been deleted from OECD test guidelines in 2014. Another in vivo genotoxicity assay, that is, “Mouse *Dlb-1* mutation assay”, detects mutations at the *Dlb-1* locus in colon, which determines the expression of the binding site for the lectin *Dolichos biflorus* agglutinin [11]. C57BL/6J × SWR F1 mice are exposed to chemicals and the mutants are detected as clones of epithelial cells not stained with a peroxidase conjugated with the agglutinin. The assay is capable of identification of mutagens in colon but is not applicable to other organs such as liver.

To circumvent the above limitations, transgenic mice for mutagenesis have been developed in late 1980’s and 1990’s. Big Blue mice, Muta Mice and *gpt* delta mice are representative transgenic mice for mutagenesis and they use lambda phage as a vector having reporter genes for mutations [12–15]. The phages are recovered from the genomic DNA of mice by in vitro lambda phage packaging reactions and in vivo mutations are detected after introduction of the rescued phage to indicator *Escherichia coli* (*E. coli*). Because the vector DNA having the reporter genes is recovered from the mouse genome to bacteria, they are called shuttle vectors. Although the reporter genes are bacteria or phage origin, the assays allow detection of mutations in any organ of mice such as liver, lung, bone marrow or testis. In addition, DNA sequence analysis can reveal mutation spectra associated with chemical exposure. About 10 years later from the development of transgenic mice, transgenic rats were developed because rats are more frequently used for cancer bioassays. Currently Big Blue rats having lambda LIZ and *gpt* delta rats having lambda EG10 are commercially available and widely used for in vivo mutagenesis [7, 16, 17]. Therefore, we focus on these two in vivo assays and discuss what has been revealed by the assays (Table 1). In the later part of this review, we review several transgenic rat models for chemical carcinogenesis (Table 2) and discuss the future perspective.

**Table 1:** Summary of experimental data of transgenic rat models for mutagenesis

Agents	Animal	Dose	Route	Sex	Age (weeks)	Administration time/Sampling time (days)	Tissue	Transgenic assays	Positive response	Reference
Acetaminophen	*gpt* delta rat (SD)	10000 ppm	Diet	Female	11	91/91	Liver	*gpt*	-	[167]
Acrylamide	Big Blue rat (F344)	1.4 mM	Drinking water	Male	7	60/60	Liver	*cII*	-	[168]
							Bone marrow	*cII*	-	Ibid
							Thyroid	*cII*	-	Ibid
							Testis	*cII*		Ibid
	*gpt* delta rat (F344)	0.7 mM	Drinking water	Female	7	60/60	Liver	*cII*	-	Ibid
							Bone marrow	*cII*	+	Ibid
							Thyroid	*cII*	+	ibid
							Mammary gland	*cII*	-	Ibid
				Male	7	60/60	Liver	*cII*	-	Ibid
				Female	7	60/60	Liver	*cII*	-	Ibid
		80 ppm	Drinking water	Male	3	28/28	Testis	*gpt*	+	[169]
							Liver	*gpt*	-	Ibid
		40 ppm					Testis	*gpt*	-	Ibid
							Liver	*gpt*	-	Ibid
		20 ppm					Testis	*gpt*	-	Ibid
							Liver	*gpt*	-	Ibid
		80 ppm	Drinking water	Male	11	28/28	Testis	*gpt*	-	Ibid
							Liver	*gpt*	-	Ibid
		40 ppm					Testis	*gpt*	-	Ibid
		20 ppm					Liver	*gpt*	-	Ibid
							Testis	*gpt*	-	Ibid
							Liver	*gpt*	-	ibid
Aflatoxin B1	Big Blue rat (F344)	0.5 mg/kg	Gavage	Male	13	1/14	Liver	*lacI*	+	[105]
				Female	13	1/14	Liver	*lacI*	+	Ibid
		0.5 mg/kg	Gavage	Female	6–8	1/14	Liver	*lacI*	+	[170]
								*cII*	+	[109]
		0.25 mg/kg	Intraperitoneal	Male	13–19	1/14	Liver	*lacI*	+	[18]
2-Amino-3-methylimidazo[4,5-*f*]quinoline (IQ)	Big Blue rat (F344)	20 mg.kg	Gavage	Male	5	1/14	Liver	*lacI*	+	[171]
							Colon	*lacI*	+	Ibid
							Kidney	*lacI*	+	Ibid
		20 mg/kg x5	Gavage	Male	5	5/18	Liver	*lacI*	+	Ibid
							Colon	*lacI*	+	Ibid
							Kidney	*lacI*	+	Ibid
		200 ppm	Diet	Male	8	21/21	Liver	*cII*	+	[172]
							Colon	*cII*	+	Ibid
		70 ppm	Diet	Male	8	21/21	Liver	*cII*	+	Ibid
							Colon	*cII*	+	Ibid
					9–12	21/21	Liver	*cII*	+	[100]
							Colon	*cII*	+	Ibid
		20 ppm	Diet	Male	8	21/21	Liver	*cII*	-	[172]
							Colon	*cII*	-	Ibid
	*gpt* delta rat (SD)	300 ppm	Diet	Female	11	91/91	Liver	*gpt*	+	[167]
2-amino-3,8-dimethylimidazo[4,5-*f*]quinoxaline (MeIQx)	Big Blue rat (F344)	100 ppm	Diet	Male	6	112/112	Liver	*lacI*	+	[85]
							Colon	*lacI*	+	Ibid
							Zymbal gland	*lacI*	+	Ibid
							Kidney	*lacI*	-	Ibid
							Spleen	*lacI*	-	Ibid
							Lung	*lacI*	-	Ibid
							Testis	*lacI*	-	Ibid
							Heart	*lacI*	-	Ibid
							Brain	*lacI*	-	Ibid
							Fat tissue	*lacI*	-	Ibid
							Skeletal muscle	*lacI*	-	Ibid
		10 ppm	Diet	Male	6	112/112	Liver	*lacI*	+	Ibid
		1 ppm	Diet	Male	6	112/112	Liver	*lacI*	-	Ibid
		0.1 ppm	Diet	Male	6	112/112	Liver	*lacI*	-	Ibid
		0.01 ppm	Diet	Male	6	112/112	Liver	*lacI*	-	Ibid
		0.001 ppm	Diet	Male	6	112/112	Liver	*lacI*	-	Ibid
2-Amino-1-methyl-6-phenylimidazo(4,5-*b*)pyridine (PhIP)	Big Blue rat (F344)	100 mg/kg, bw	Intraperitoneal	Male	8+22	1/14	Colon	*lacI*	+	[102]
				Female	8+22	1/14	Colon	*lacI*	+	Ibid
		75 mg/kg ×10	Oral	Female	7	12/54	Mammary gland	*lacI*	+	[40]
					22	12/54	Mammary gland	*lacI*	+	Ibid
		70 mg/kg ×12	Gavage	Male	13–15	28/30	Liver	*cII*	-	[41]
							Kidney	*cII*	-	Ibid
							Prostate	*cII*	+	Ibid
							Seminal vesicle	*cII*	+	Ibid
							Colon	*cII*	+	Ibid
							Spleen	*cII*	+	Ibid
		70 mg/kg ×24	Gavage	Male	13–15	56/60	Liver	*cII*	-	Ibid
							Kidney	*cII*	-	Ibid
							Prostate	*cII*	+	Ibid
							Seminal vesicle	*cII*	+	Ibid
							Colon	*cII*	+	Ibid
							Spleen	*cII*	+	Ibid
		400 ppm	Diet	Male	6	60/67	Colon mucosa	*lacI*	+	[23, 44]
								*cII*	+	[23]
				Female	6	60/67	Colon mucosa	*lacI*	+	[23, 44]
								*cII*	+	[23]
		200 ppm	Diet	Male	7	61/68	Distal colon	*lacI*	+	[45, 173]
							Proximal colon	*lacI*	+	Ibid
							Cecum	*lacI*	+	Ibid
							Prostate	*lacI*	+	[42]
				Female	7	61/68	Distal colon	*lacI*	+	[45]
							Proximal colon	*lacI*	+	Ibid
							Cecum	*lacI*	+	Ibid
		100 ppm	Diet	Male and Female	7	47/54	Distal colon	*lacI*	+	[174]
							Cecum	*lacI*	+	Ibid
				Male	7	47/54	Kidney	*lacI*	+	[98]
				Female	7	47/54	Kidney	*lacI*	+	Ibid
	Big Blue rat (F344 × SD)F1	65 mg/kg ×10	Gavage	Female	6	12/350–441	Mammary gland	*lacI*	+	[39]
Amosite asbestos	Big Blue rat (F344)	2 mg/rat	Intratracheal	Male	11	1/28	Lung	*lacI*	-	[103, 126]
						1/112	Lung	*lacI*	+	Ibid
		2 mg/rat ×4	Intratracheal	Male	11	28/56	Lung	*lacI*	-	Ibid
						28/133	Lung	*lacI*	+	Ibid
		1 mg/rat	Intratracheal	Male	11	1/28	Lung	*lacI*	-	Ibid
						1/112	Lung	*lacI*	-	Ibid
Aristolochic acid	Big Blue rat (F344)	10 mg/kg	Gavage	Male	6	84/84	Kidney	*cII*	+	[112, 113]
							Liver	*cII*	+	[112]
							Testis	*cII*	-	[175]
		1.0 mg/kg	Gavage	Male	6	84/84	Kidney	*cII*	+	[112, 113]
							Liver	*cII*	+	[112]
		0.1 mg/kg	Gavage	Male	6	84/84	Kidney	*cII*	+	[112, 113]
							Liver	*cII*	-	[112]
	Big Blue rat (F344)	10 mg/kg	Gavage	Male	6	84/84	Spleen	*cII*	+	[176]
		1.0 mg/kg	Gavage		6	84/84	Spleen	*cII*	+	Ibid
		0.1 mg/kg	Gavage		6	84/84	Spleen	*cII*	-	Ibid
	*gpt* delta rat (F344)	1 mg/kg	Gavage	Male	7	28/31	Kidney	*gpt*	+	[177]
		0.3 mg/kg	Gavage		7	28/31	Liver	*gpt*	+	Ibid
Benzo[*a*]pyrene (BP)	Big Blue rat (F344)	100 mg/kg, bw	Intraperitoneal	Male	8+22	1/14	Liver	*lacI*	+	[102]
				Female	8+22	1/14	Liver	*lacI*	+	Ibid
		5 mg/rat	Intraperitoneal	Female	10	1/28	Omentum	*lacI*	+	[178]
						1/84	Omentum	*lacI*	+	Ibid
						1/168	Omentum	*lacI*	+	Ibid
	*gpt* delta rat (SD)	125 mg/kg	Intraperitoneal	Male	9–10	1/7	Liver	*gpt*	+	[17]
								Spi^–^	+	Ibid
		62.5 mg/kg	Intraperitoneal	Male	9–10	1/7	Liver	*gpt*	+	Ibid
								Spi^−^	-	Ibid
Bitumen fumes	Big Blue rat (F344)	6 hr ×5	Inhalation	Male	8	5/35	Lung	*cII*	-	[132]
										Ibid
*N*-butyl-*N*-(4-hydroxybutyl)nitrosamine	Big Blue rat (F344)	0.05 %	Drinking water	Male	8	14/42	urothelial cells (urinary bladder)	*cII*	+	[179]
							smooth muscle cells (urinary bladder)	*cII*	-	Ibid
							Ureter	*cII*	-	Ibid
							Kidney	*cII*	-	Ibid
							Liver	*cII*	-	Ibid
						42/70	urothelial cells (urinary bladder)	*cII*	+	Ibid
							smooth muscle cells (urinary bladder)	*cII*	+	Ibid
							Ureter	*cII*	-	Ibid
							Kidney	*cII*	-	Ibid
							Liver	*cII*	-	Ibid
Citrinin	*gpt* delta rat (F344)	40 mg/kg (decreased to 30 mg/kg at day 4)	Gavage	Male		28/31	Kidney	*gpt*	-	[65]
		20 mg/kg				28/31	Kidney	*gpt*	-	Ibid
Comfrey	Big Blue rat (F344)	2 %	Diet	Male	6	84/84	Liver	*cII*	+	[180]
Conjugated linoleic acid	Big Blue rat (F344)	1 %(w/w)	Diet	Male and Female	7	54/61	Distal colon	*lacI*	-	[174]
							Cecum	*lacI*	-	Ibid
							Kidney	*lacI*	-	[98]
Crocidolite	Big Blue rat (F344)	5 mg/rat	Intraperitoneal	Female	10	1/28	Omentum	*lacI*	-	[178]
						1/84	Omentum	*lacI*	+	Ibid
						1/168	Omentum	*lacI*	+	Ibid
		2 mg/rat	Intraperitoneal	Female	10	1/28	Omentum	*lacI*	-	Ibid
						1/84	Omentum	*lacI*	-	Ibid
						1/168	Omentum	*lacI*	-	Ibid
Cyproterone acetate	Big Blue rat (F344)	160 mg/kg	Gavage	Female	12	1/14	Liver	*lacI*	+	[90]
		100 mg/kg	Gavage	Female	12	1/1	Liver	*lacI*	+	Ibid
						1/2	Liver	*lacI*	+	Ibid
						1/3	Liver	*lacI*	+	Ibid
						1/7	Liver	*lacI*	+	Ibid
						1/14	Liver	*lacI*	+	Ibid
						1/28	Liver	*lacI*	+	Ibid
						1/42	Liver	*lacI*	+	Ibid
						1/56	Liver	*lacI*	+	Ibid
		80 mg/kg	Gavage	Female	12	1/14	Liver	*lacI*	+	Ibid
		40 mg/kg	Gavage	Female	12	1/14	Liver	*lacI*	+	Ibid
		20 mg/kg	Gavage	Female	12	1/14	Liver	*lacI*	+	Ibid
		10 mg/kg	Gavage	Female	12	1/14	Liver	*lacI*	+	Ibid
		5 mg/kg	Gavage	Female	12	1/14	Liver	*lacI*	-	Ibid
		5 mg/kg ×21	Gavage	Female	11–12	21/22	Liver	*lacI*	+	[181]
		1.0 mg/kg ×21	Gavage	Female	11–12	21/22	Liver	*lacI*	-	Ibid
		0.2 mg/kg ×21	Gavage	Female	11–12	21/22	Liver	*lacI*	-	Ibid
Daidzein	Big Blue rat (F344)	1 g/kg	Diet	Female	7	112/112	Mammary gland	*lacI*	-	[95]
							Uterus	*lacI*	-	[96]
		0.25 g/kg	Diet	Female	7	112/112	Mammary gland	*lacI*	-	[95]
							Uterus	*lacI*	-	[96]
	Big Blue rat (F344), ovariectomized	1 g/kg	Diet	Female	7	112/112	Uterus	*lacI*	-	[96]
		0.25 g/kg	Diet	Female	7	112/112	Uterus	*lacI*	-	Ibid
Diallyl sulfide (DAS)	Big Blue rat (F344)	200 mg/kg ×2	Gavage	Male	7–10	7/21	Esophagus	*lacI*	-	[93]
2,4-diaminotoluene (2,4-DAT)	*gpt* delta rat (F344)	30 mg/kg	Gavage	Male	7	28/31	Liver	*gpt*	+	[55]
		10 mg/kg	Gavage	Male	7	28/31	Liver	*gpt*	+	Ibid
	*gpt* delta rat (F344)	500 ppm	Diet	Male	7	91/91	Liver	*gpt,* Spi^−^	+	[7]
		500 ppm	Diet	Male	7	91/91	Kidney	*gpt*	-	Ibid
		250 ppm	Diet	Male	7	91/91	Liver	*gpt,* Spi^−^	+	Ibid
		250 ppm	Diet	Male	7	91/91	Kidney	*gpt*	-	Ibid
		125 ppm	Diet	Male	7	91/91	Liver	*gpt*	+	Ibid
		125 ppm	Diet	Male	7	91/91	Liver	Spi-	-	Ibid
		125 ppm	Diet	Male	7	91/91	Kidney	*gpt*	-	Ibid
2,6-diaminotoluene (2.6-DAT)	*gpt* delta rat (F344)	60 mg/kg	Gavage	Male	7	28/31	Liver	*gpt*	-	[55]
	*gpt* delta rat (F344)	500 ppm	Diet	Male	7	91/91	Liver	*gpt,* Spi^−^	-	[7]
		500 ppm	Diet	Male	7	91/91	kideny	*gpt*	-	Ibid
1,2,3,4-Diepoxybutane	Big Blue rat (F344)	3.8 ppm, 6 hr ×5×2	Inhalation	Female and Male	6–8	14/49	Spleen	*lacI*	-	[182]
						14/28	Bone marrow	*lacI*	+	Ibid
Diesel exhaust (DE)	Big Blue rat (F344)	6 mg SPM/m^3^, 12 hr/day	Inhalation	Male	6	28/31	Lung	*lacI*	+	[130]
		1 mg SPM/m^3^, 12 hr/day	Inhalation	Male	6	28/31	Lung	*lacI*	-	ibid
		80 mg DEP/kg	Diet	Male	8	21/21	Lung	*cII*	-	[131]
		20 mg DEP/kg	Diet	Male	8	21/21	Lung	*cII*	-	Ibid
		8 mg DEP/kg	Diet	Male	8	21/21	Lung	*cII*	-	Ibid
		2 mg DEP/kg	Diet	Male	8	21/21	Lung	*cII*	-	Ibid
		0.8 mg DEP/kg	Diet	Male	8	21/21	Lung	*cII*	-	Ibid
		0.2 mg DEP/kg	Diet	Male	8	21/21	Lung	*cII*	-	Ibid
Di(2-ethyl-hexyl)phthalate (DEHP)	*gpt* delta rat (SD)	12000 ppm	Diet	Female	11	91/91	Liver	*gpt*	-	[167]
								Spi^*−*^	-	Ibid
Diethylnitrosamine	*gpt* delta rat (F344)	20 mg/kg (once a week)	Intraperitoneal	Male	7	91/91	Liver	*gpt,* Spi^−^	+	[7]
				Male	7	91/91	kidney	*gpt*	+	ibid
5-*p*-Dimethylaminophenylazobenzthiazole (5BT)	Big Blue rat (F344)	10 mg/kg ×10	Gavage	Male	12	10/20	Liver	*lacI*	+	[61]
		0.03 %	Diet	Male	12	10/20	Liver	*lacI*	+	ibid
6-*p*-Dimethylaminophenylazobenzthiazole (6BT)	Big Blue rat (F344)	10 mg/kg ×10	Gavage	Male	12	10/20	Liver	*lacI*	+	[61, 183]
		0.03 %	Diet	Male	12	10/20	Liver	*lacI*	+	ibid
7,12-Dimethylbenzanthracene (DMBA)	Big Blue rat (F344)	130 mg/kg	Gavage	Female	7	1/14	Splenic lymphocyte	*lacI*	+	[27]
						1/14	Mammary gland	*lacI*	+	[184]
						1/14	Bone marrow	*lacI*	+	[26]
						1/42	Splenic lymphocyte	*lacI*	+	[27]
						1/42	Mammary gland	*lacI*	+	[184]
						1/42	Bone marrow	*lacI*	+	[26]
						1/70	Splenic lymphocyte	*lacI*	+	[27]
						1/70	Mammary gland	*lacI*	+	[18]
						1/70	Bone marrow	*lacI*	+	[26]
						1/98	Splenic lymphocyte	*lacI*	+	[27]
						1/98	Mammary gland	*lacI*	+	[184]
						1/98	Bone marrow	*lacI*	+	[26]
		80 mg/kg	Gavage	Female	7	1/112	Liver	*cII*	+	[94]
							Uterus	*lacI*	+	[96]
		75 mg/kg	Gavage	Female	7	1/14	Splenic lymphocyte	*lacI*	-	[27]
						1/42	Splenic lymphocyte	*lacI*	+	Ibid
						1/70	Splenic lymphocyte	*lacI*	+	Ibid
						1/98	Splenic lymphocyte	*lacI*	+	Ibid
						1/126	Splenic lymphocyte	*lacI*	-	Ibid
		20 mg/kg	Gavage	Female	7	1/14	Splenic lymphocyte	*lacI*	-	[27]
						1/14	Mammary gland	*lacI*	-	[184]
						1/14	Bone marrow	*lacI*	+	[26]
						1/42	Splenic lymphocyte	*lacI*	+	[27]
						1/42	Mammary gland	*lacI*	-	[184]
						1/42	Bone marrow	*lacI*	+	[26]
						1/70	Splenic lymphocyte	*lacI*	-	[27]
						1/70	Mammary gland	*lacI*	-	[184]
						1/70	Bone marrow	*lacI*	+	[26]
						1/98	Splenic lymphocyte	*lacI*	-	[27]
						1/98	Mammary gland	*lacI*	-	[18])
						1/98	Bone marrow	*lacI*	+	[26]
						1/126	Splenic lymphocyte	*lacI*	-	[27]
						1/126	Mammary gland	*lacI*	-	[184]
	Big Blue rat (F344), ovariectomized	80 mg/kg	Gavage	Female	7	1/112	Heart	*lacI*	+	[185]
							Liver	*cII*	+	[94]
							Uterus	*lacI*	+	[96]
Dimethylnitrosamine (DMN)	Big Blue rat (F344)	6.0 mg/kg ×9	Gavage	Male	12	11/12	Liver	*lacI*	+	[20]
								*cII*	+	Ibid
		2.0 mg/kg ×9	Gavage	Male	12	11/12	Liver	*lacI*	+	Ibid
								*cII*	+	Ibid
		0.6 mg/kg ×9	Gavage	Male	12	11/12	Liver	*lacI*	-	Ibid
								*cII*	-	Ibid
		0.2 mg/kg ×9	Gavage	Male	12	11/12	Liver	*lacI*	-	Ibid
								*cII*	-	Ibid
	Big Blue rat (F344)	25 ppm	Drinking water			14/14	Liver	*cII*		[186]
		10 ppm	Drinking water			14/14	Liver	*cII*		Ibid
		5 ppm	Drinking water			14/14	Liver	*cII*		Ibid
		1 ppm	Drinking water			14/14	Liver	*cII*		Ibid
		0.1 ppm	Drinking water			14/14	Liver	*cII*		Ibid
Ellagic acid	Big Blue rat (F344)	4 g/kg	Diet	Male	7–10	42/42	Esophagus	*lacI*	-	[93]
1,2-Epoxy-3-butene	Big Blue rat (F344)	29.9 ppm, 6 hr ×5×2	Inhalation	Female	6–8	14/49	Spleen	*lacI*	-	[182]
						14/28	Bone marrow	*lacI*	+	Ibid
Estragole	*gpt* delta rat (F344)	600 mg/kg	Gavage	Male	5	5 days per week x 4 weeks	Liver	*gpt*	(72) toxic but 2 of 5 survived rats showed positive responses	
		200 mg/kg	Gavage	Male	5	5 days per week x 4 weeks	Liver	*gpt*	+	ibid
		66 mg/kg	Gavage	Male	5	5 days per week x 4 weeks	Liver	*gpt*	-	Ibid
		22 mg/kg	Gavage	Male	5	5 days per week x 4 weeks	Liver	*gpt*	-	Ibid
*N*-ethyl-*N*-nitrosourea (ENU)	Big Blue rat (F344)	100 mg/kg	Intraperitoneal	Male	8+22	1/14	Liver	*lacI*	+	[102]
				Female	8+22	1/14	Liver	*lacI*	+	Ibid
	*gpt* delta rat (SD)	100 mg/kg	Intraperitoneal	Male	5	1/7	Liver	*gpt*	+	[17]
								Spi^−^	-	Ibid
		50 mg/kg ×5	Intraperitoneal	Male	10	5/7	Liver	*gpt*	+	Ibid
						5/21	Liver	*gpt*	+	Ibid
						5/35	Liver	*gpt*	+	Ibid
						5/70	Liver	*gpt*	+	Ibid
17beta-estradiol	Big Blue rat (F344)	0.005 g/kg	Diet	Female	7	112/112	Mammary gland	*lacI*	-	[95]
							Uterus	*lacI*	-	[96]
	Big Blue rat (F344), ovariectomized	0.005 g/kg	Diet	Female	7	112/112	Heart	*lacI*	-	[95]
							Uterus	*lacI*	-	[96]
Ethanol	Big Blue rat (F344)	5 %	Drinking water	Male	7–10	21/21	Esophagus	*lacI*	-	[93]
Fructose	Big Blue rat (F344)	30 %	Diet	Male	9–12	35/35	Liver	*cII*	-	[101]
							Colon	*cII*	+−	Ibid
Furan	Big Blue rat (F344)	30 mg/kg	Gavage	Male	7	56/56	Liver	*cII*	-	[187]
		16 mg/kg	Gavage	Male	7	56/56	Liver	*cII*	-	Ibid
		8 mg/kg	Gavage	Male	7	56/56	Liver	*cII*	-	Ibid
		2 mg/kg	Gavage	Male	7	56/56	Liver	*cII*	-	Ibid
Genistein	Big Blue rat (F344)	1 g/kg	Diet	Female	7	112/112	Mammary gland	*lacI*	-	[95]
					7	112/112	Liver	*cII*	-	[94]
							Uterus	*lacI*	-	[96]
		0.25 g/kg	Diet	Female	7	112/112	Mammary gland	*lacI*	-	[95]
							Uterus	*lacI*	-	[96]
	Big Blue rat (F344), ovariectomized	1 g/kg	Diet	Female	7	112/112	Heart	*lacI*	-	[185]
							Uterus	*lacI*	-	[96]
							Liver	*cII*	-	[94]
		0.25 g/kg	Diet	Female	7	112/112	Heart	*lacI*	-	[185]
							Uterus	*lacI*	-	[96]
Glass wool fibres	Big Blue rat (F344)	2 mg/rat	Intratracheal	Male	11	1/28	Lung	*lacI*	-	[127, 188]
						1/112	Lung	*lacI*	-	Ibid
		2 mg/rat x4	Intratracheal	Male	11	28/56	Lung	*lacI*	-	Ibid
						28/133	Lung	*lacI*	-	Ibid
		1 mg/rat	Intratracheal	Male	11	1/28	Lung	*lacI*	-	Ibid
						1/112	Lung	*lacI*	-	Ibid
Glucose	Big Blue rat (F344)	30 %	Diet	Male	9–12	35/35	Liver	*cII*	-	[101]
							Colon	*cII*	+−	Ibid
Glycidamide	Big Blue rat (F344)	1.4 mM	Drinking water	Male	7	60/60	Liver	*cII*	-	[168]
							Bone marrow	*cII*	+	Ibid
							Thyroid	*cII*	+	Ibid
							Testis	*cII*	-	Ibid
				Female	7	60/60	Liver	*cII*	-	Ibid
							Bone marrow	*cII*	+	Ibid
							Thyroid	*cII*	+	Ibid
							Mammary gland	*cII*	-	Ibid
		0.7 mM	Drinking water	Male	7	60/60	Liver	*cII*	-	Ibid
				Female	7	60/60	Liver	*cII*	-	Ibid
Hexavalent chromium [Cr(VI)] (as sodium dichromate dihydrate)	Big Blue rat (F344)	180 ppm (as Cr(VI))	Drinking water	Male	10	28/31	Oral cavity (gingival/bubbal)	*cII*	-	[189]
							Oral cavity (gingival/palate)	*cII*	-	Ibid
*N*-hydroxy-2-acetylaminofluorene	Big Blue rat (F344)	25 mg/kg ×1	Intraperitoneal	Male	6	1/42	Liver	*lacI*	+	[28, 190]
		25 mg/kg ×2	Intraperitoneal	Male	6	5/42	Liver	*lacI*	+	Ibid
		25 mg/kg ×4	Intraperitoneal	Male	6	13/42	Liver	*lacI*	+	Ibid
							Spleen lymphocytes	*lacI*	+	Ibid
4-Hydroxy-PCB3 (4-OH-PCB3)	Big Blue rat (F344)	82 mg/kg ×4	Intraperitoneal	Male	6	22/38	Liver	*lacI*	+−	[133]
							Lung	*lacI*	+−	[135]
alpha-Hydroxytamoxifen	Big Blue rat (F344)	54 μmol/kg ×21	Intraperitoneal	Female	8	21/51	Liver	*cII*	+	[22]
							Liver	*lacI*	+	[191]
							Uterus	*lacI*	-	Ibid
		0.103 mmol/kg ×10	Gavage	Female	6–8	10/56	Liver	*lacI*	+	[192]
Lard	Big Blue rat (F344)	30 %	Diet	Male	8	21/21	Liver	*cII*	-	[193]
							Colon	*cII*	-	Ibid
		10 %	Diet	Male	8	21/21	Liver	*cII*	-	Ibid
							Colon	*cII*	-	Ibid
		3 %	Diet	Male	8	21/21	Liver	*cII*	-	Ibid
							Colon	*cII*	-	Ibid
Leucomalachite green	Big Blue rat (F344)	543 ppm	Diet	Female	6	28/28	Liver	*lacI*	-	[79]
						112/112	Liver	*lacI*	-	[79, 81]
						224/224	Liver	*lacI*	-	[79]
		272 ppm	Diet	Female	6	28/28	Liver	*lacI*	-	Ibid
						112/112	Liver	*lacI*	-	Ibid
						224/224	Liver	*lacI*	-	Ibid
		91 ppm	Diet	Female	6	112/112	Liver	*lacI*	-	Ibid
*N*-Methyl-amyl-nitrosamine	Big Blue rat (Sprague Dawley/Big Blue F1)	25 mg/kg ×10	Intraperitoneal		10	64/70	Esophagus	*lacI*	+	[194]
3-Methylcholanthrene	Big Blue rat (F344)	80 mg/kg ×4	Intraperitoneal	Male	6	22/38	Liver	*lacI*	+	[133]
							Lung	*lacI*	+	[135]
4-Monochlorobiphenyl (PCB3)	Big Blue rat (F344)	113 mg/kg ×4	Intraperitoneal	Male	6	22/38	Liver	*lacI*	+	[133]
							Lung	*lacI*	+	[135]
Madder color and lucidin-3-O-primeveroside (LuP)	*gpt* delta rat (F344)	Madder color 5.0 % w/w	Diet	Male	5	56/56	Kidney	*gpt,* Spi^−^	+	[73]
		LuP 0.3 % w/w	Diet	Male	5	56/56	Kidney	*gpt,* Spi^−^	+	Ibid
3-MCPD	*gpt* delta rat (F344)	40 mg/kg	Gavege	Male	6	5 times per week x 4 weeks/1	Kidney and testis	*gpt,* Spi^−^	-	[66]
		the equimolar esters	Gavege	Male	6		Kidney and testis	*gpt,* Spi^−^	-	Ibid
Methyleugenol	*gpt* delta rat (F344)	100 mg/kg	Gavage	Male and female	5	91/91	Liver	*gpt,* Spi^−^	+	[74]
		30 mg/kg	Gavage	Male and female	5	91/91	Liver	*gpt,* Spi^−^	-	Ibid
		10 mg/kg	Gavage	Male and female	5	91/91	Liver	*gpt,* Spi^−^	-	Ibid
Nickel subsulfide (Ni_3_S_2_)	Big Blue rat (F344)	6 mg/kg (130 mg/m^3^), 2 h	Inhalation	Male	9	1/14	Lung	*lacI*	-	[123]
							Nasal mucosa	*lacI*	-	Ibid
	*gpt* delta rat (F344)	1 mg/animal	Intratracheally instillation	Male	12	28/28	Lung	*gpt,* Spi^−^	-	[124]
		0.5 mg/animal	Intratracheally instillation	Male	12	28/28	Lung	*gpt,* Spi^−^	-	Ibid
		1 mg/animal	Intratracheally instillation	Male	12	90/90	Lung	*gpt,* Spi^−^	-	ibid
		0.5 mg/animal	Intratracheally instillation	Male	12	90/90	Lung	*gpt,* Spi^−^	-	Ibid
*N*-nitrosomethylbenzylamine	Big Blue rat (F344)	2 mg/kg ×2	Subcutaneous	Male	7–10	7/21	Esophagus	*lacI*	+	[93]
6-Nitrochrysene	Big Blue rat (F344 × SD)F1	100 μmole/rat x 8	Oral	Female	5	50/274	Mammary gland	*cII*	+	[195]
		50 μmole/rat x 8	Oral	Female	5	50/274	Mammary gland	*cII*	+	Ibid
*N*-nitrosopyrrolidine	*gpt* delta rat (SD)	200 ppm	Drinking water	Female	11	91/91	Liver	*gpt*	+	[167]
4-nitroquinoline-1-oxide	Big Blue rat (F344)	10 ppm	Drinking water	Male	10–11	28/31	Oral cavity (gingival/bubbal)	*cII*	+	[196]
							Oral cavity (gingival/palate)	*cII*	+	Ibid
							Liver	*cII*	-	Ibid
							Bone marrow	*cII*	-	Ibid
	Big Blue rat (F344)	10 ppm	Drinking water	Male	10	28/31	Oral cavity (gingival/bubbal)	*cII*	+	[189]
							Oral cavity (gingival/palate)	*cII*	+	Ibid
Ochratoxin A	*gpt* delta rat (F344)	5 ppm	Diet	Male and female	5	28/28	outer medulla of kidney	*gpt*	-	[50]
				Male and female	5	28/28	outer medulla of kidney	Spi^−^	+	Ibid
				Male and female	5	91/91	whole kidney	*gpt,* Spi^−^	-	ibid
		5 ppm	Diet	Male	5	28/28	outer medulla of kidney	Spi^−^	+	[65]
Phenacetin	*gpt* delta rat (SD)	0.50 %	Diet	Male and female	7	182/182	Kidney	*gpt,* Spi^−^	-	[52]
				Male and female	7	182/182	Liver	*gpt*	+	Ibid
				Male and female	7	182/182	Liver	Spi^−^	-	ibid
				Male	7	364/364	Kidney	*gpt*	+	Ibid
				Male	7	364/364	Kideney	Spi^−^	-	Ibid
				Female	7	364/364	Kideney	*gpt,* Spi^−^	-	Ibid
				Male	7	364/364	Liver	*gpt,* Spi^−^	+	Ibid
				Female	7	364/364	Liver	*gpt,* Spi^−^	+	Ibid
Potassium Bromate (KBrO_3_)	Big Blue rat (F344)	500 ppm	Drinking water	Male	6	112/112	Kidney	*lacI*	+	[87]
		125 ppm	Drinking water	Male	6	112/112	Kidney	*lacI*	-	Ibid
		30 ppm	Drinking water	Male	6	112/112	Kidney	*lacI*	-	Ibid
		8 ppm	Drinking water	Male	6	112/112	Kidney	*lacI*	-	Ibid
		2 ppm	Drinking water	Male	6	112/112	Kidney	*lacI*	-	Ibid
		0.2 ppm	Drinking water	Male	6	112/112	Kidney	*lacI*	-	Ibid
		0.02 ppm	Drinking water	Male	6	112/112	Kidney	*lacI*	-	Ibid
		0.002 ppm	Drinking water	Male	6	112/112	Kidney	*lacI*	-	Ibid
	*gpt* delta rat (SD)	500 ppm	Drinking water	Male	5	7/7	Kidney	*gpt*	-	[119]
								Spi^−^	-	Ibid
						35/35	Kidney	*gpt*	-	Ibid
								Spi^*−*^	-	Ibid
						63/63	Kidney	*gpt*	-	Ibid
								Spi^*−*^	+	Ibid
						91/91	Kidney	*gpt*	-	Ibid
								Spi^−^	+	Ibid
		250 ppm	Drinking water	Male	5	91/91	Kidney	*gpt*	-	Ibid
								Spi^−^	-	Ibid
		125 ppm	Drinking water	Male	5	91/91	Kidney	*gpt*	-	Ibid
								Spi^−^	-	Ibid
		60 ppm	Drinking water	Male	5	91/91	Kidney	*gpt*	-	Ibid
								Spi^−^	-	Ibid
	*gpt* delta rat (F344)	500 ppm	Drinking water	Male	5	63/63	Kidney	*gpt*	-	[120]
								Spi^−^	-	Ibid
				Female	5	63/63	Kidney	*gpt*	+	Ibid
								Spi^−^	-	Ibid
Riddelliine	Big Blue rat (F344)	1.0 mg/kg ×5 ×12	Gavage	Female	6	82/83	Liver	*cII*	+	[115]
		0.3 mg/kg ×5 ×12	Gavage	Female	6	82/83	Liver	*cII*	+	ibid
					6	82/83	Liver parenchymal cells	*cII*	-	[116]
					6	82/83	Liver endothelial cells	*cII*	+	Ibid
		0.1 mg/kg ×5 ×12	Gavage	Female	6	82/83	Liver	*cII*	+	[115]
Rock wool fibers	Big Blue rat (F344)	2 mg/rat	Intratracheal	Male	11	1/28	Lung	*lacI*	-	[127, 188]
						1/112	Lung	*lacI*	+	Ibid
		2 mg/rat ×4	Intratracheal	Male	11	28/56	Lung	*lacI*	-	Ibid
						28/133	Lung	*lacI*	+	ibid
		1 mg/rat	Intratracheal	Male	11	1/28	Lung	*lacI*	-	Ibid
						1/112	Lung	*lacI*	+	Ibid
Selenium (as sodium selenite) with dimethylhydrazine (DMH) injection	Big Blue rat (F344)	2 μg Se/g diet with DMH injection	Diet			63–84/63–84 (DMH (25 mg/kg body weight, i.p., weekly, twice) was injected after 3 weeks of the diet.	Liver	*cII*	+	[197]
							Colon	*cII*	+	Ibid
		0.2 μg Se/g diet with DMH injection	Diet			63–84/63–84 (DMH (25 mg/kg body weight, i.p., weekly, twice) was injected after 3 weeks of the diet.	Liver	*cII*	+	Ibid
							Colon	*cII*	+	Ibid
									*No effect of Se.	
Sucrose	Big Blue rat (F344)	30 %	Diet	Male	9–12	35/35	Liver	*cII*	-	[101]
							Colon	*cII*	+−	Ibid
		34.50 %	Diet	Male	8–12	21/21	Liver	*cII*	-	[136]
							Colon	*cII*	+	ibid
		13.80 %	Diet	Male	8–12	21/21	Liver	*cII*	-	Ibid
							Colon	*cII*	+	Ibid
		6.90 %	Diet	Male	8–12	21/21	Liver	*cII*	-	Ibid
							Colon	*cII*	-	Ibid
		13.40 %	Diet	Male	9–12	21/21	Liver	*cII*	-	[100]
							Colon	*cII*	+	Ibid
		3.45 %	Diet	Male	9–12	21/21	Liver	*cII*	-	Ibid
							Colon	*cII*	+	Ibid
2,3,7,8-tetrachlorodibenzo-p-dioxin (TCDD)	Big Blue rat (F344)	2 μig/kg ×12	Gavage	Male	8	42/56	Liver	*lacI*	-	[128]
				Female	8	42/56	Liver	*lacI*	-	Ibid
Tamoxifen	Big Blue rat (F344)	54 μmol/kg ×21	Intraperitoneal	Female	8	21/51	Liver	*cII*	+	[22]
							Liver	*lacI*	+	[191]
							Uterus	*lacI*	-	Ibid
		20 mg/kg ×42	Gavage	Female	6–8, 16–18	42/56	Liver	*lacI*	+	[170]
								*cII*	+	[109]
					6–8	42/42	Liver	*lacI*	+	[110]
						42/56	Liver	*lacI*	+	Ibid
						42/126	Liver	*lacI*	+	Ibid
						42/210	Liver	*lacI*	+	Ibid
		10 mg/kg ×42	Gavage	Female	6–8	42/56	Liver	*lacI*	+	[170]
								*cII*	+−	[109]
	*gpt* delta rat (F344)	40 mg/kg	Gavege	Female		21/21	Liver	*gpt,* Spi^−^	+	[58]
			Gavege	Female		21/21	Kidney	*gpt,* Spi^−^	-	Ibid
		20 mg/kg	Gavege	Female		21/21	Liver	*gpt,* Spi^−^	+	Ibid
			Gavege	Female		21/21	Kidney	*gpt,* Spi^−^	-	Ibid
		500 ppm	Diet	Female		91/91	Liver	*gpt,* Spi^−^	+	Ibid
			Diet	Female		91/91	Kidney	*gpt,* Spi^−^	-	Ibid
		250 ppm	Diet	Female		91/91	Liver	*gpt,* Spi^−^	+	Ibid
			Diet	Female		91/91	Kidney	*gpt,* Spi^−^	-	Ibid
Thiotepa Toremifene	Big Blue rat (F344)	1.4 mg/kg ×12	Intraperitoneal	Male	7	28/35	Spleen lymphocytes	*lacI*	+	[29]
	Big Blue rat (F344)	20 mg/kg ×42	Gavage	Female	6–8	42/56	Liver	*lacI*	-	[170]
	*gpt* delta rat (F344)	40 mg/kg	Gavege	Female		21/21	Liver	*gpt,* Spi^−^	-	[58]
			Gavege	Female		21/21	Kidney	*gpt,* Spi^−^	-	Ibid
Tris(2,3-dibromopropyl)phosphate (TDBP)	Big Blue rat (F344)	2000 ppm	Diet	Male	6–8	45/45	Kidney (outer medulla)	*lacI*	+	[47]
							Kidney (inner medulla)	*lacI*	+	Ibid
							Kidney (cortex)	*lacI*	+	Ibid
		100 ppm	Diet	Male	6–8	45/45	Kidney (outer medulla)	*lacI*	+	Ibid
							Kidney (inner medulla)	*lacI*	+	Ibid
							Kidney (cortex)	*lacI*	+	Ibid
Uracil	Big Blue rat (F344)	3 %	Diet	Male	6	14/14	Bladder	*lacI*	-	[25]
						70/70	Bladder	*lacI*	+	Ibid
						140/140	Bladder	*lacI*	+	Ibid
						357/357	Bladder	*lacI*	+	Ibid

**Table 2:** Summary of transgenic rat models for carcinogenesis

Appellation	Transgene	Feature	Target organ	Usage	Reference
Hras128	carrying three copies of the human c-Ha-*ras* proto-oncogene, including its own promoter region	Highly susceptible to chemical-induced carcinogenesis	mammary gland, esophagus,bladder	carcinogenesis mechanisms, screening for chemo preventive agents	[138]
TRAP	the simian virus 40 (SV40) large T antigen under probasin promoter control	Males demonstrate atypical epithelial cell proliferation in the prostate from 4 weeks of age and develop prostate carcinomas at 100 % incidence before they are 15 weeks old	prostate	carcinogenesis mechanisms, screening for chemo preventive agents	[145]
Cx32Δ Tg	a dominant negative mutant of the *connexin* 32 gene under albumin promoter control	The gap junctional intercellular communications were disrupted in the liver and highly susceptible to chemical-induced hepatocarcinogenesis.	liver	carcinogenesis mechanisms	[150]
alb-SV40 Tag Transgenic Rat	promoter-enhancer sequences of the mouse albumine gene linked 5' to the simian virus-40 T antigen gene	All animal exhibit focal lesions and nodules in liver at 4 months of age. These lesions were GST-P negative.	liver	mechanism study of spontaneous hepatocarcinogenesis in this transgenic rats	[198]
Hras250	human Ha-*ras* ^G12V^ oncogene regulated by the Cre/lox system	targeted activation of a human oncogenic-ras transgene in rat pancreas induce pancreatic ductal adenocarcinomas	Pancreas	carcinogenesis mechanisms	[153]
Kras327	human K-ras^G12V^ oncogene regulated by the Cre/lox system	targeted activation of a human oncogenic-ras transgene in rat pancreas induce pancreatic ductal adenocarcinomas	Pancreas	carcinogenesis mechanisms	[152]

## Transgenic rats for mutagenesis

Before establishment of transgenic rats for mutagenesis, there was a gap between in vivo genotoxicity assays and rodent cancer bioassays in terms of animal species. In vivo genotoxicity assays such as chromosome aberration test and micronucleus test have been conducted more frequently with mice than with rats because of the ease of handling and clearer genetic background. In contrast, rodent cancer bioassays have been conducted with rats more frequently than mice because of the lower frequency of spontaneous tumors and larger body mass. This species difference leads to discrepancy of test results between mice in mutagenesis and rats in carcinogenesis. Aflatoxin B_1_ gives negative or weakly positive results in genotoxicity with mice while rats give strong positives in carcinogenicity assays [18]. To fill in the gap, transgenic rats have been engineered. Nowadays, they are used as a standard tool to examine the mutagenicity of chemicals in the target organs of carcinogenesis.

### Assay systems

Although both Big Blue rats and *gpt* delta rats use lambda phage as vectors of reporter genes, the assay systems are different as described below.

#### Big blue rats

**Fig. 1:**
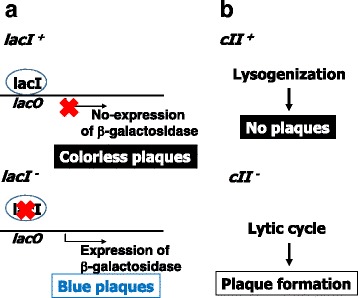
Mutant selections for Big Blue rats. **a** *lacI* selection. When LacI, the repressor protein of the *lac* operon, is active, it represses the expression of beta-galactosidase, which leads to colorless plaques. When the *lacI* gene is inactivated by mutations, beta-galactosidase is expressed, which leads to blue plaques. **b** *cII* selection. The cII protein is the critical switch in the lytic/lysogenic cycles of lambda phage. It activates the expression of the lambda *cI* (repressor) and *int* (integrase) genes, which are required for the establishment of lysogeny. The cII protein is negatively regulated by host *E. coli* Hfl protease, which digests the cII protein. In the *hfl* ^*-*^ background, the cII level is high, and therefore the lambda becomes lysogen. Only *cII* mutants can enter a lytic cycle and make plaques at 24 °C. The *cI* ^-^ mutants can’t enter the lytic cycle at this temperature. Therefore, the *cII* selection for Big Blue rats is conducted at 24 °C

Big Blue rats were generated by microinjection of lambda LIZ phage DNA into fertilized eggs of Fischer 344 (F344) rats [16]. In addition, the embryonic fibroblasts, i.e., Rat 2 cells, were established for an in vitro transgenic assay [19]. Originally, color selection with *lacI* was adopted for mutant detection but later more convenient *cII* selection was applied to Big Blue rat assays [20] (Fig. 1a, b). The gene *lacI* encodes a repressor protein LacI, which suppresses the expression of beta-galactosidase in *E. coli*. Therefore, inactivation of *lacI* by mutations results in the expression of beta-galactosidase and production of blue plaque in the presence of X-gal, while wild-type *lacI* leads to colorless plaques. However, this selection is time consuming and expensive because X-gal is an expensive chromogenic agent. In contrast, the CII protein induces the expression of the *cI* and the *int* genes that are required for a phage lysogeny [21]. In the *hfl* ^*−*^ *E. coli*, phages with active *cII* gene can’t enter a lytic cycle and form no plaques because of the deficient in Hfl protease. This protease degrades CII protein and lets the phage enter a lytic cycle. The only phages with inactive *cII* mutants can make plaques with the *E. coli hfl* ^*−*^ cells. Thus, this is a positive selection, and much more convenient and less expensive than the original *lacI* assay. The coding size of *lacI* is 1080 bp while that of *cII* is 294 bp, which makes *cII* more attractive for determination of mutation spectrum.

Because *cII* was introduced several years after the original *lacI* color selection has been established, the level of spontaneous mutations and sensitivity to chemically-induced mutagenesis were compared between the reporter genes. Chen et al. [22] report that spontaneous mutation frequency of *cII* in liver is markedly higher than that of *lacI* (80 × 10^−6^ vs 10 × 10^−6^). Stuart et al. [23] also report that the mutation frequency of *cII* in colon mucosa is higher than that of *lacI* (78 × 10^−6^ vs 23 × 10^−6^). The *cII* gene has six G:C base pairs between nucleotide number 179 and 185, which is one of the hot spots of spontaneous mutagenesis. The high background makes smaller fold increases in mutation frequency after chemical treatments with alpha-hydroxytamoxifen and tamoxifen [22]. However, Gollapudi et al. [20] report that there is no significant difference in spontaneous and dimethyl nitrosamine (DMN)-induced mutation frequencies in liver between *cII* and *lacI* of Big Blue rats (99 × 10^−6^ vs 85 × 10^−6^ for spontaneous and 415 × 10^−6^ vs 400 × 10^−6^ for DMN.)

In both *lacI* and *cII*, deamination of 5-methylcytosine (5-MeC), which results in G:C to A:T transitions, is a major source of spontaneous mutations. Full methylation of *cII* and *lacI* in Big Blue rats is reported in bone marrow, bladder, liver, spleen and breast [24]. Spontaneous *lacI* mutation frequencies are lower in bone marrow and bladder compared to liver, which can’t be explained by the status of methylation of 5-MeC [25, 26]. Monroe et al. [24] suggest, therefore, that other mechanisms besides deamination of 5-MeC contribute to spontaneous mutagenesis in Big Blue system.

Because *lacI* is not an endogenous gene but a bacterial gene, the sensitivity of *lacI* and an endogenous gene, i.e., *Hprt*, in spleen was compared in Big Blue rats. Both genes were responded to 7, 12-dimethylbenz[*a*]anthracene (DMBA) [26, 27], *N*-hydroxyacetylaminofluorene [28] and thiotepa, an anticancer drug [29], and the mutation frequencies were increased. However, spontaneous mutation frequencies of *Hprt* were about 10 times lower than those of *lacI* [27]. Thus, the fold increases were larger in *Hprt* than in *lacI*. For example, the mutation frequency of *Hprt* was increased more than 10 fold by thiotepa treatments (3.5 × 10^−6^ vs 41.1 × 10^−6^) while that of *lacI* was increased about four fold by the same treatment (34.8 × 10^−6^ vs 140.9 × 10^−6^) [29]. In addition, the mutation spectra were different where *Hprt* recovered a fraction of large deletions not found among *lacI* mutants [29].

In summary, *lacI* and *cII* can be regarded as effective surrogate genes for in vivo mutations while spontaneous mutation frequency of *cII* may be higher than that of *lacI*. Caution should be payed that deletion mutations may be missed by the surrogate genes.

#### *gpt* delta rats

**Fig. 2:**
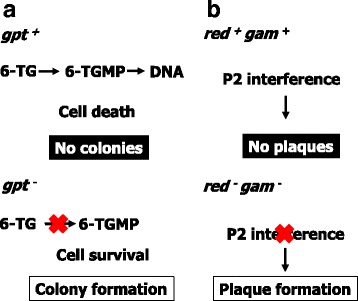
Mutant selection for *gpt* delta rats. **a** *gpt* selection. The *E. coli gpt* gene encodes guanine phosphoribosyl transferase, which attaches a phosphoribose to 6-TG. The phosphoribosylated 6-TG is further phosphorylated and finally incorporated into DNA. Incorporation of 6-TG is toxic to *E. coli* and cell death is induced. Therefore, only when the *gpt* gene is inactivated by mutations, *E. coli* can make colonies on a plate containing 6-TG. **b** Spi^-^ selection. The wild-type lambda phages lyse *E. coli*, thereby making phage plaques. However, if the *E. coli* chromosome harbors P2 phage DNA, which is called P2 lysogen, the wild-type lambda phage can’t lyse P2 lysogen. Only the defective lambda phage whose *red* and *gam* genes are inactivated can lyse P2 lysogen. The resulting plaques are called P2 plaques. Because the *red* and *gam* genes are localized in lambda genome side by side, the inactivation of two genes are most likely induced by deletions in the region

*gpt* delta rats were generated by microinjection of lambda EG10 DNA into fertilized eggs of Sprague-Dawley (SD) rats [17]. The SD *gpt* delta rats were later crossed with F344 rats for 15 generations, thereby establishing F344 *gpt* delta rats [7]. Two distinct selection systems are available for *gpt* delta mice and rats (Fig. 2a). One is *gpt* selection for detection of point mutations and the other is Spi^-^ selection for deletions [15, 30]. The *gpt* gene is a bacterial counterpart of *Hprt* and encodes guanine phosphoribosyl transferase. When the *gpt* gene is inactivated by mutations, the *E. coli* host cells possessing plasmid carrying mutated *gpt* gene can survive on plates containing 6-thioguanine (6-TG) while those harboring plasmid carrying the wild-type *gpt* gene die because they phosphoribosylate 6-TG and incorporate 6-TGMP into DNA. Therefore, the *gpt* selection is a positive selection.

Spi^-^ stands for sensitive to P2 interference [31] (Fig. 2b). This selection allows selective detection of deletion mutants of lambda phage. In wild-type *E. coli*, the wild-type lambda phage lyses the *E. coli*, thereby forming phage plaques. However, if *E. coli* chromosome possesses P2 phage DNA, that is called P2 lysogen, the wild-type lambda phage can’t form plaques. This phenomenon is called “P2 interference”. However, when two genes of lambda phage, i.e., the *red* and *gam* genes, are simultaneously inactivated, the defective phage can make plaques in P2 lysogen. The plaques are called Spi^-^ plaques. Since the *red* and *gam* genes are located side by side in the lambda DNA, the simultaneous inactivation of two genes are most likely induced by deletion of the region containing the two genes. The unique feature of Spi^-^ selection is specific detection of deletion mutations including frameshift mutations.

The transgene lambda EG10 having the *gpt* gene and the *red/gam* genes is located in the chromosome four of *gpt* delta rats. The exact location of the integration site in the rat genome was determined by next generation DNA sequencer (NGS) [32]. About 72 kb genomic sequence was deleted during integration of the transgene and smaller genetic rearrangements were also induced by the integration. Unlike *gpt* delta mice, which have lambda EG10 in both chromosome 17, *gpt* delta rats are heterozygous where lambda EG10 is integrated in only one allele of chromosome 4. This is because homozygous *gpt* delta rats are defective in tooth development and can’t survive after weaning. Specific PCR primers that can be used to amplify the DNA sequence between rat chromosome and the integrated lambda EG10 are available. They can be used to distinguish between wild-type rats and *gpt* delta rats. The average spontaneous *gpt* and Spi^-^ mutant frequencies in liver are 4.5 × 10^−6^ and 2.7 × 10^−6^, respectively [33]. The frequencies are significantly lower than those of the *lacI* and *cII* genes. The low spontaneous mutant frequencies of *gpt* and Spi^-^ are similar to those of *gpt* delta mice.

### Issues that have been examined by transgenic rat assays

#### Organ/tissue specificity

An important feature of chemical carcinogens is the organ specificity. They induce cancer in specific organs, which are called target organs for carcinogenesis. Aflatoxin B_1_, aristolochic acid and *o*-toluidine are all potent human carcinogens but they induce cancer in different organs, i.e., liver by aflatoxin B_1_, kidney by aristolochic acid and bladder by *o*-toluidine [34–36]. Thus, an interesting question for transgenic rat assays for mutagenesis is whether mutations can be identified in the target organs for carcinogenesis.

2-Amino-1-methyl-6-phenylimidazo[4,5-*b*]pyridine (PhIP) is a heterocyclic amine in cooked food and administration of PhIP in diet causes cancer in the prostate in male rats and in the mammary glands in females [37, 38]. It was examined, therefore, whether PhIP induces mutations in the target organs in a sex specific manner. PhIP-induced mutations were identified in mammary glands of female rats [39, 40] and prostate in males [41, 42]. These results suggest the causal link between mutagenesis and carcinogenesis induced by PhIP in mammary glands and prostates. However, mutations in prostate were identified not only in ventral prostate where cancer is induced but also in dorsolateral and anterior lobe where cancer is sparingly induced [41]. This raised a question as to what factors define the lobe specificity of PhIP-induced carcinogenesis. Interestingly, PhIP acts as a promoter and induces cell proliferation only in the ventral prostate [41]. Thus, PhIP may be an organ- and lobe-specific promoter while it acts as an initiator in all three lobes

PhIP induces colon cancer much more frequently in male rats than in females [43]. Therefore, colon mutations were examined in male and female Big Blue rats. It was revealed that mutations were almost equally induced in both sexes [44, 45]. The mutation spectra induced by PhIP were also similar in both sexes, i.e., one base deletions including the guanine deletion at 5′-GGGA-3′ [44]. These results suggest that factors other than mutagenesis strongly contribute to PhIP-induced carcinogenesis and also that the factors may determine the sex-specific induction of colon cancer by PhIP.

The relationship between mutagenesis and carcinogenesis has been examined even at the sub-organ level as in the case of PhIP in the prostate. Tris(2,3-dibromopropyl)phosphate (TDBP) induces tumors specifically in outer medulla in the kidney of rats [46]. Mutations were examined in the inner medulla, outer medulla and cortex of kidney, and the mutation frequency was in the order of cortex followed by outer medulla (the target site) and inner medulla [47]. The highest mutation induction does not coincide with the localization of tumors. However, cell proliferation is increased specifically in the outer medulla after TDBP treatment [46, 48]. Thus, it was concluded that combined effects of cell proliferation and induction of mutations are responsible for sub-organ-specific tumor formation by TDBP.

Ochratoxin A, a mycotoxin, also induces renal tumors in rats specific in S3 segment of the proximal tubules [49]. Unlike TDBP, mutations are induced only in the outer medulla, which is primarily occupied by the S3 segment of the proximal tubules [50]. No mutations were detected in the cortex. Thus in this case, specific induction of mutations in outer medulla might account for the sub-organ-specific induction of tumors in rats (See more in Genotoxic versus non-genotoxic carcinogens section).

Phenacetin, an analgesic drug, induces tumors in kidney but not in liver [51]. The in vivo mutagenesis in kidney and liver was examined with SD *gpt* delta rats fed with diet containing phenacetin for 26 and 52 weeks [52]. Mutations were detected in both kidney and liver and the mutation frequency was much higher in liver (non-target organ) than in kidney (target organ). The results suggest the intensity of mutagenicity does not necessarily correlate with the induction of tumor formation.

#### Carcinogens versus structurally-related non-carcinogens

Chemical carcinogens excert the adverse effects depneding on the chemical structures. Even the structures are similar, their carcinogenicity is sometimes completely different. Transgenic rats for mutagenesis were examined for their ability to distinguish mutagenicity of structural isomers, i.e., one is a carcinogen and the other is a non-carcinogen. 2,4-Diaminotoluene (2,4-DAT) is an intermediate in chemical industry but induces hepatic tumors in male and female rats and mammary and subcutaneous tumors in female rats [53]. The isomer 2,6-DAT is an intermediate of dyes and rubber chemicals and is not carcinogenic in rats and mice despite the structural similarity to 2,4-DAT [54]. Interestingly, both DATs are mutagenic in Ames Salmonella strains [7], suggesing the potential mutagenicity of both chemicals. The in vivo mutagenicity of 2,4-DAT and 2,6-DAT was examined in liver and kidney of male *gpt* delta rats [7]. The rats were fed 2,4-DAT or 2,6-DAT in diet for 13 weeks and the mutations were examined. Only 2,4-DAT induced *gpt* and Spi^-^ mutations in liver but not in kidney. 2,6-DAT was negative in *gpt* and Spi^-^ assays in liver and kideny. The results suggest that in vitro mutagenicity should be carefully examined by in vivo mutagenicty assay. The mutagenicity of 2,4-DAT but not 2,6-DAT in liver of *gpt* delta rats was also reported by 4 weeks administration of gavage [55].

Tamoxifen is a nonsteroid antiestrogen that is used as adjuvant therapy for breast cancer. However, tamoxifen is carcinogenic in liver in rats [56]. The structural analogue toremifen is not carcinogenic [57]. To examine whether transgenic rats distinguish two compounds in terms of mutagenesis, female F344 *gpt* delta rats were treated with either tamoxifen or toremifen [58]. Tamoxifen significantly enhanced *gpt* and Spi^-^ mutation frequencies in the liver. The treatment did not increase the mutation frequencies in the kidney, a non-target organ for carcinogenesis. Toremifen did not increase *gpt* and Spi^-^ mutation frequencies in liver and kidney. The results clearly indicate that tamoxifen is mutagenic in the target organ for carcinogenesis but the strustural analogue toremifen is not.

6-*p*-Dimethylaminophenylazobenzthiazole (6BT) is a potent liver carcinogen in rats [59]. It induces malignant liver tumors after 2-to-3 months of dietary administration in a riboflavin-deficient diet. In contrast, the analogue 5-*p*-dimethylaminophenylazobenzthiazole (5BT) gives no tumors after 6 month administration. Both chemicals are potent mutagens in Ames Salmonella strains [60]. The mutagenicity of 6BT and 5BT was examined with Big Blue rats and unexpectedly both were mutagenic in liver [61]. Thus, mutagenicity did not account for the marked difference of the carcinogenicity of two closely-related compounds. It is speculated that differential cell proliferation effects on oval cells in liver may explain the difference. 6BT induces the proliferation of ovall cells by either gavage or in diet while 5BT is inactive in this respect. Oval cells may be progenitor cells for hepatocellular carcinoma [62].

#### Genotoxic versus non-genotoxic carcinogens

A key question for evaluation and regulation of chemical carcinogens is whether mutations are involved in the mechanisms of carcinogenesis. If the chemical induces mutations in the target organ, thereby causing carcinogenesis, the chemical is classified as “a genotoxic carcinogen”, which has no threshold or safety dose for the action [4]. In contrast, when the chemical dose not induce mutations in the target organ despite the carcinogenicity, the chemical is classified as “a non-genotoxic carcinogen”, which has threshold or safety dose and can be used in the society below the safety dose. If the chemical is judged as a genotoxic carcinogen, the chemical is not be considered acceptable for use as food additives, pesticides or veterinary drugs [63, 64].

Several carcinogenic compounds in food were examined for the mutagenicity in the target organs for carcinogenesis with *gpt* delta rats [63]. It was revealed that citrinin and 3-monochloropropane-1,2-diol (3-MCPD) were negative, and hence they were classified as non-genotoxic carcinogens [65, 66]. Citrinin is a food-contaminated mycotoxin and induces renal tumors in rats [67]. It may induce tumors via cell cycle progression but not genotoxicity [65]. 3-MCPD is regarded as a rat renal and testicular carcinogen [68] and is mutagenic in *Salmonella* and *E. coli* strains for mutagenicity assays [69]. The fatty acid esters of 3-MCPD are generated during food processing and exert renal toxicity [70]. The esters are metabolized to 3-MCPD in vivo [71]. Because of the negative mutagenicity in vivo, 3-MCPD and the fatty acid esters are judged as non-genotoxic carcinogens [66]. On the other side, estragole [72], madder color [73] and methyleugenol [74] were positive in the transgenic assay and thus mutagenicity may participate in the carcinogenesis. Estragole is a natural organic compound and frequently used as a flavoring food additive, but is carcinogenic in liver of mice [75]. Despite the in vivo mutagenicity, estragole is not mutagenic in *Salmonella* and *E. coli* strains for mutagenicity assays [76]. Madder color is a dye and a potent carcinogen in kidney and liver in rats [77], and thus its use as a food additive has been banned in Japan in 2004. Methyleugenol is a fragrance and flavoring agent but is a hepatocarcinogen in F344 rats [78].

Malachite green is a dye that has been widely used as an antifungal agent in fish industry, and leucomalachite green is a reduction product and a major metabolite of malachite green [79]. Malachite green induces adenoma and/or carcinoma in thyroid gland, liver and mammary gland of female F344 rats and leucomalachite green induces adenoma in the testis of male rats [80]. Female Big Blue rats were fed leucomalachite green for 4, 16 or 32 weeks and mutations were analyzed in *Hprt* in spleen, micronucleus formation in bone marrow and *lacI* mutation in liver [81]. No increases were observed in *Hprt* mutation frequency and micronucleus formation. About three fold increases in *lacI* mutant frequency were observed in rats treated for 16 weeks [79]. DNA adduct levels increased in liver of rats. However, the following mutation spectrum analysis indicated that the apparent increase in mutation frequency was due to expansion of spontaneous mutations [81]. It is still enigmatic how malachite green and leucomalachite green induce tumors in rats.

Ochratoxin A [49], a mycotoxin, is an interesting agent because it induces Spi^-^ mutations but not *gpt* [50, 82]. It induces Spi^-^ mutations in the target site of carcinogenesis, i.e., the outer medulla of kidney, when male *gpt* delta rats were treated with ochratoxin A. Large deletions with the size of more than 1 kb are induced by the treatment. Experiments with p53 deficient *gpt* delta mice suggest that Spi^-^ mutant frequency, but not *gpt*, was increased by ochratoxin A treatment [83, 84]. No mutagenicity was observed in p53 proficient mice. It appears that double-strand breaks in DNA are induced in the target site of kidney of rats, which leads to large deletions. It is puzzling, however, why *gpt* mutations are not induced. When DNA is damaged, *gpt* mutations are usually more frequently induced compared to Spi^-^ mutations. If ochratoxin A induces DNA adducts, it should induce *gpt* mutations as well as Spi^-^ mutations. It is tempting to speculate, therefore, that ochratoxin A may interact with proteins involved in DNA replication, repair or chromosome segregation, thereby inducing double-strand breaks in DNA. If so, ochratoxin A may not be a genotoxic carcinogen although it induces mutations in the target organ of carcinogenesis.

#### Threshold or low dose effects

Although it is supposed that genotoxic carcinogens have no thresholds or safety level, the following experiments exhibit no effective dose levels for in vivo mutations of genotoxic carcinogens. Male Big Blue rats were fed a diet containing 0.001, 0.01, 0.1, 1, 10 or 100 ppm of 2-amino-3,8-dimethylimidazo[4,5-*f*]quinoxaline (MeIQx) for 16 weeks and the *lacI* mutation frequency and glutathione S-transferase placental form (GST-P) positive foci in the liver were examined [85]. MeIQx is a heterocyclic amine formed during cooking and induces liver tumors in rats [86]. The mutation frequencies significantly increased at doses of 10 and 100 ppm, and GST-P positive foci significantly increased at a dose of 100 ppm. No statistical increases in both frequencies were observed, however, at lower doses, indicating the existence of no effective doses for mutagenesis and carcinogenesis.

Similarly, male Big Blue rats were administered with potassium bromate (KBrO_3_) in drinking water at concentrations of 0, 0.02, 0.2, 2, 8, 30, 125 and 500 ppm for 16 weeks [87]. The *lacI* mutation in the kidney was induced only at a concentration of 500 ppm. No mutagenicity was detected at 125 ppm or lower doses. Histopathological changes in renal tubular cells were observed at doses of 125 and 500 ppm but not at 30 ppm or lower doses. 8-oxoguanine in DNA was formed only at a dose of 500 ppm. KBrO_3_ is an oxidizing agent and used as a maturing agent for flour and as a dough conditioner [68]. However, it induces renal cell tumors in male and female rats after oral administration for 2 years in the drinking water [88]. The results suggest that there may be safety dose for the genotoxic carcinogen.

Cyproterone acetate (CPA) is an antiandrogenic drug that is used for women in long term treatments of excel androgen levels. However, it induces liver tumors in rats [89]. Female Big Blue rats were treated with CPA at a single dose of 0, 5, 10, 20, 40, 80 and 100 mg/kg and the *lacI* mutation frequency was determined in the liver 2 weeks after the last treatment. Significant increase in mutation frequency was observed at a dose of 10 mg/kg or higher, and no mutations were induced at a dose of 5 mg/kg [90]. Because high amounts of DNA adducts were formed at the non-effective dose of 5 mg/kg, it was assumed that the mitotic activity required for conversion of DNA adducts to mutation was not sufficiently strong at the dose.

Collectively, these results suggest the existence of no-effective dose for mutagenesis in the target organs for carcinogenesis even for mutagenic carcinogens. It remains uncertain, however, the sensitivity to detect the mutations is high enough to analyze the subtle increase in mutation frequencies. It is suggested that no-effective levels for mutagenesis vary depending on the in vivo models and also that the lower no-effective levels are detected with lower spontaneous mutation frequencies [91]. To detect the no-effective levels, mathematical models such as Points of Departure (PoD) have been proposed [92].

#### Multiple exposure or chemoprevention

Genotoxic effects of chemicals are sometimes enhanced or attenuated by dietary supplements. In addition, people are exposed to multiple chemicals in real life. Therefore, they may exert additive or synergistic effects on the genotoxic effects. Transgenic rats for mutagenesis have been utilized to examine the combined genotoxic effects of more than one chemical in vivo.

Ellagic acid, green tea and diallyl sulfide (DAS) were examined for the chemo preventive effects against *N*-nitrosomethylbenzylamine (NMBA)–induced mutations in the esophagus of Big Blue rats [93]. Addition of ellagic acid in diet, replacing drinking water with green tea or gavage of DAS significantly reduced the mutagenicity of NMBA. In contrast, 5 % ethanol to the drinking water enhanced the mutagenicity.

Endogenous estrogen status and addition of genistein, a phytoestrogen, were examined for the modulating effects on DMBA-induced mutation in liver of Big Blue rats [94]. Ovariectomized female rats exhibited higher mutation frequencies than the intact rats, suggesting the endogenous ovarian hormones may have an inhibitory effect on liver mutagenesis by DMBA. Dietary supplement of genistein in the ovariectomized and the intact rats did not alter the spontaneous and induced mutations in liver. Ovariectomized female Big Blue rats were also used to examine the modulating effects of daidzein, genistein and 17-beta-estradiol on DMBA-induced mutagenesis in the mammary glands [95] and uterus [96]. Daidzein and genistein are major constituents of isoflavones and interact with the alpha and beta estrogen receptors in the mammary glands. Daidzein, genistein and 17-beta-estradiol each did not significantly change DMBA-induced mutagenesis in the mammary glands and uterus.

Conjugated linoleic acid is a mixture of heat-derivatives of linoleic acid, and is shown to be protective against heterocyclic amine-induced carcinogenesis [97]. Antimutagenic effects of conjugated linoleic acid was examined in kidney of male and female Big Blue rats treated with PhIP [98]. Conjugated linoleic acid reduced PhIP-induced mutations of female rats but not those of male rats. Therefore, the protective effects are sex-dependent.

High intake of sucrose is associated with increased risk of colon cancer [99]. Co-mutagenic effects of sucrose were examined in colon of 2-amino-3-methylimidazo[4,5-*f*]quinoline (IQ)-treated Big Blue rats [100]. Sucrose and IQ increased the mutation frequencies and the combined treatment with sucrose and IQ was additive, indicating that sucrose and IQ induce mutations independently. It is worth noticing that sucrose is mutagenic in vivo [101], which will be discussed more detail below (Sweet diet section). On the other hand, dietary restriction may delay aging and age-related diseases. The effects of dietary restriction on PhIP-induced mutation in the distal colon were examined [102]. However, the restriction did not alter the mutation frequency in male and female Big Blue rats. To examine the interactions between tobacco smoking and asbestos exposure, Big Blue rats were exposed to benzo[*a*]pyrene (BP) and amosite intratracheally and mutations were analyzed in the lung of Big Blue rats. Combined instillation of amosite and BP exhibited a highly significant synergistic effect [103]. The mutation frequency of BP was enhanced more than two times when combined with amosite, which was not mutagenic in lung.

The compound 2,3,7,8-tetrachlorodibenzo-*p*-dioxin (TCDD) is an environmental contaminant and a potent carcinogen in laboratory rodents [104]. Modulating effects of TCDD on mutagenesis was examined with male and female Big Blue rats [105]. The rats were pre-exposed to TCDD for 6 weeks (2 μg twice per week) and then they were given aflatoxin B_1_ at a dose of 0.5 mg/kg by gavage. After 2 weeks, the *lacI* mutation frequency was measured. TCDD pre-treatments did not significantly modulate the mutation frequency in male. However, the female mutation frequency was reduced to the control level. DNA sequence analysis confirmed the absence of aflatoxin B_1_-induced transversion mutations in female rats. It is speculated that sex-specific factors such as estrogens or estrogen receptors may play a role in the sex-dependent chemopreventive effects of TCDD against aflatoxin B_1_-induced mutagenesis.

#### Tamoxifen

As described in Carcinogens versus structurally-related non-carcinogens, tamoxifen is widely used for adjuvant therapy in the breast cancer patient for many years. However, tamoxifen induces endometrial cancer in women, and liver and endometrial tumors in rats [106]. There is no evidence, however, that tamoxifen induces liver tumors in humans. Tamoxifen is metabolically activated to alpha-hydroxytamoxifen, which is further acitvated by sulfotransferase and finally induces DNA adducts. Rat sulfotransferase activates alpha-hydroxytamoxifen but human enzyme does not [107]. This may be the reason for the species difference between human and rat for liver tumorigenesis by tamoxifen. Because tamoxifen is inactive in a battery of short-term tests for mutagenesis [108], the in vivo mutagenicity was examined with Big Blue rats and *gpt* delta rats. Tamoxifen induced *lacI*, *cII*, *gpt* and Spi^-^ mutations in the liver, mainly G:C to T:A transversions and −1 frameshift [58, 108–110]. Alpha-hydroxytamoxifen also induces mutations in the liver with the spectrum of mutation of G:C to T:A [22]. Thus, it appears that tamoxifen induces liver tumors in rats via alpha-hydroxytamoxifen-induced mutagenesis.

#### Naturally occurring carcinogens

Several plant constituents often used for herbal treatments were examined for the mutagenicity in vivo because of the carcinogenicity in experimental animals and in humans. Aristolochic acid is a nephrotoxin and carcinogenic in kidney and forestomach in rodents [111]. It has been associated with the development of urothelial cancer in humans. Male Big Blue rats were gavaged with aristolochic acid for 3 months, and the DNA adduct levels and mutations were examined in liver (a non-target organ) and kidney (a target organ) [112, 113]. Kidney exhibited at least two fold higher levels of DNA adducts and mutations than liver. A:T to T:A transversions were the predominant mutation in both organs. In this case, higher DNA damage and mutation frequencies were observed in the target organ than in the non-target organ.

Riddelliine is a naturally occurring pyrrolizidine alkaloid that induces liver hemangiosarcomas in rats and mice [114]. Female Big Blue rats were gavaged with riddelliine for 12 weeks and the mutations were analyzed in liver [115]. Mutations were induced in a dose-dependent manner and the major mutation was G:C to T:A. Later, liver was dissected into parenchymal and endothelial cells and riddelliine-induced mutations were analyzed in the cells [116]. Mutation was specifically induced in the endothelial cells but not in the parenchymal cells. Because hemangiosarcomas are derived from endothelial cells, the results indicate a good correlation between mutagenesis and carcinogenesis at a cell-type level.

#### Oxidative damage

Oxidative stress is an important factor for in vivo mutagenesis and carcinogenesis. Although KBrO_3_ induces 8-oxoguanine in DNA, which leads to G:C to T:A mutations, in vitro genotoxicity assays suggest that KBrO_3_ induces deletions rather than G:C to T:A transversions [117, 118]. Male SD *gpt* delta rats were given KBrO_3_ in drinking water for 13 weeks and the level of 8-oxoguanine in DNA and mutations were analyzed in the kidney [119]. Increases of 8-oxoguanine in DNA occurred after 1 week treatment at 500 ppm. Spi^-^ mutations were increased after 9 weeks administration at 500 ppm but no significant increases in mutation frequency were observed at 500 ppm earlier than 9 weeks. No *gpt* mutations were observed even at week 13. The results suggest that deletions but not G:C to T:A are induced by KBrO_3_ in kideny of rats and also that 9 weeks may be necessary to convert the induced 8-oxoguanine in DNA to mutations. It is worth noticing, however, that male Big Blue rats (F344) exhibited mainly G:C to T:A transversions in kideny when they were treated with KBrO_3_ in drinking water at 500 ppm for 16 weeks [87] (see Threshold or low dose effects). Different genetic background of rats (SD versus F344) might affect the spectrum of mutations. When female F344 *gpt* delta rats were given KBrO_3_ in drinking water at 500 ppm for 9 weeks, *gpt* mutation frequency was significantly increased along with slight increase of Spi^-^ mutations [120]. However, the spectrum of induced *gpt* mutations was not predominated by G:C to T:A but various types of mutations including −1 frameshift were observed. Thus, it remains to be clarified what types of mutations are induced by KBrO_3_ in vivo.

#### DNA non-reactive carcinogens (metals, asbestos and TCDD)

Several nickel compounds are carcinogenic in humans and animals [121]. Nickel subsulfide (Ni_3_S_2_) is one of them and induces lung tumors in F344 rats following inhalation exposure [122]. Although Ni_3_S_2_ increased *lacI* mutation frequency in in vitro Rat2 cells, it did not enhance *lacI* mutation in the lung and nasal mucosa of male Big Blue rats when the rats were treated by inhalation through the nose [123]. Male F344 *gpt* delta rats were also treated with Ni_3_S_2_ by intratracheal instillation, but no increases in *gpt* and Spi^-^ mutant frequencies were observed in the lung [124].

Asbestos is a well-known human carcinogen that induces mesothelioma and lung cancer in exposed persons [125]. Male Big Blue rats were given amosite asbestos by intratracheal instillation with single doses of 1 or 2 mg/animal, or 4 weekly doses of 2 mg [126]. The in vivo mutations were analyzed at 4 weeks or 16 weeks after the last treatment. The average length of amosite was more than 20 μm and the average thickness of the fiber was 0.7 micron, leading to persistent presence in the lung. About two fold induction of *lacI* mutations was observed in the lung after 16 weeks exposure possibly because of the persistent inflammation induced by the treatment. Similarly, two asbestos substituent mineral fibers, i.e., rock (stone) wool RW1 and glass wool MMVF10, were examined for the in vivo mutagenicity with male Big Blue rats [127]. The man-made fibers were given to the rats by intratracheal instillation with single doses of 1 or 2 mg/animal, or 4 weekly doses of 2 mg. Exposure of RW1 for 16 weeks increased *lacI* mutant frequency about two-fold in the lung but MMVF10 did not. Because RW1 induces mild inflammation in the lung, the mutagenicity may be due to DNA damage induced by the inflammation.

TCDD induces various tumors in rats [104]. Male and female Big Blue rats were exposed to 2 μg TCDD/kg by gavage for 6 weeks but no increase in *lacI* mutation frequency was observed in the liver of both sexes [128].

Mechanical irritation by uracil-induced urolithiasis was examined for the in vivo mutagenicity with male Big Blue rats [25]. The rats were fed 3 % uracil in the diet for 50 weeks and the *lacI* mutation frequency was determined in the bladder. About three to five fold increases in the mutation frequency were observed at weeks 10, 20 and 51. The mutation spectra were similar to those of the spontaneous mutations, i.e., G:C to A:T transitions at CpG sites. Therefore, it is suggested that the elevation of spontaneous mutations may be due to cell proliferations induced by the uracil treatment.

#### Polluted air

Diesel exhaust (DE) is a factor of air pollution and a suspected cause of lung cancer and other respiratory diseases [129]. Male Big Blue rats were exposed to 1 or 6 mg/m^3^ of DE for 4 weeks [130]. The mutant frequency in lung was increased about five times over the control level by exposure to six DE mg/m^3^ but no increases were observed with 1 mg DE/m^3^. The results clearly indicate that DE is mutagenic in rat lung. When male Big Blue rats were treated with a diet containing DE from 0 to 80 mg/kg for 3 weeks, no mutation induction was observed in the lung although DNA adducts and DNA strand breaks were observed [131]. The results suggest that inhalation exposure, but not dietary exposure, is needed to evaluate the mutagenic potential of DE in lung. Road paving workers are exposed to bitumen fumes, a complex mixture of various polycyclic aromatic amines. Big Blue rats were exposed to bitumen fumes through nose, and DNA adduct levels and mutation frequencies were examined in the lung [132]. Although DNA adducts were increased by the exposure, the mutation frequencies were not enhanced. Perhaps, cell proliferation is not fully induced by the treatment.

4-Monochlorobiphenyl (PCB3) is found in indoor and outdoor air and in food [133]. Unlike polychlorinated biphenyls, PCB3 is more readily metabolized to monohydroxy-PCBs by CYP drug metabolizing enzymes and further dihydroxy-metabolites, which can be oxidized to quinones [134]. The mutagenicity of PCB3 and the metabolite, i.e., 4-hydroxy-PCB3, were examined with male Big Blue rats [133, 135]. The rats were given PCB3 or 4-hydroxy-PCB3 by intraperitoneal injection once per week for 4 weeks. In liver and lung, the mutant frequency in PCB-3-treated rats was significantly elevated and 4-hydroxy-PCB3 induced a non-significant increase in the mutant frequency.

#### Sweet diet

Cancer incidence in colon and other organs is strongly affected by diet and life style. Intake of sucrose-rich diet was examined for the in vivo mutagenicity with Big Blue rats [136]. Male Big Blue rats were fed diet with sucrose of 3.4 % (control), 6.9, 13.8 and 34.5 % for 3 weeks without affecting the overall energy and carbohydrate intake. The *cII* mutation frequency was increased about two fold in a dose-dependent manner in the colonic mucosa but no increases in the liver. No oxidative DNA damage was increased. Later, male Big Blue rats were fed diet containing 30 % sucrose or the composed sugar, i.e., either 30 % glucose or 30 % fructose for 35 days [101]. In these experiments, however, any sugar did not significantly increase the *cII* mutations in the colon and the liver, although DNA adduct levels were increased by the diet in both organs. It is suggested that indirect effects such as alterations of chemical environment in colon may account for the apparent genotoxicity.

## Transgenic rats for carcinogenesis

In carcinogenesis study field, transgenic rats provide good models too. Rats rather than mice are more frequently used in chemical carcinogenesis studies for various reasons. For example, in the liver, GST-P has been utilized as a reliable marker for early detection of preneoplastic lesions [137]. So far, more than 30 different transgenic rats have been reported and utilized in neurosciences, endocrinology and carcinogenesis fields. Transgenic rats that are highly susceptible to carcinogens or exhibit high incidence of spontaneous neoplasm are good models for screening of chemopreventive agents and mechanism studies of carcinogenesis process.

### Human c-Ha-*ras* proto-oncogene transgenic rats (Hras128)

Hras128 carries a human c-Ha-*ras* proto-oncogene including its own promoter region. Female Hras128 is highly susceptible to breast carcinogens such as *N*-methyl-*N*-nitrosourea (MNU) and PhIP [138, 139]. These chemicals induced estrogen-independent breast tumors because they did not respond to ovariectomy [140]. Esophagus and bladder tumors were highly inducible in carcinogen–treated male Hras128 [141, 142]. This Hras128 is deposited to National BioResource Project (NBRP Rat No.0376), and available from it [143]. In addition, cell lines (RMC-1, RMC-2, RMC-3, RMC-6, RMC-11, RMC-17) derived from Hras128 mammary adenocarcinoma are also available from RIKEN cell bank [144].

### Probasin-SV40 T antigen transgenic rats (TRAP)

TRAP expresses the simian virus 40 (SV40) large T antigen under probasin promoter control. This animal was established to obtain sufficient size of samples of prostate cancer. In the male TRAP, prostate carcinomas are developed at 100 % incidence in all lobes (ventral, dorsolateral and anterior) before 15 weeks of age [145]. Since these tumors are androgen dependent, it is expected to utilize TRAP as a model for understanding the mechanisms of relapsing of tumors that are androgen independent. Chemopreventive studies and mechanism studies utilizing TRAP have been also reported [146–148].

### *Connexin 32* dominant-negative transgenic rats (Cx32Δ Tg)

Employment of the dominant negative mutants is one of the alternatives to gene targeting in rat. Cx32Δ Tg expresses a dominant negative mutant of *connexin 32* (Cx32). Cx32 is a major gap junction protein in the liver. They formed transmembrane channels between adjacent cells. In the liver of this animal, localization of normal connexins is disrupted and gap junction capacities are markedly decreased [149]. Chemical-induced carcinogenesis studies using Cx32Δtransgenic revealed that disruption of gap junctional intercellular communications in vivo resulted in hepatocarcinogenesis and its progression [150, 151]. In addition, this transgenic rat can be utilized to mechanism studies of the onset of toxicity which are related to cell-cell communications [149].

### Transgenic rats carrying a mutated H- or K-*ras* gene controlled by Cre/loxP activation (Hras250 and Kras327)

These transgenic rats express a human activated *RAS* oncogene regulated by the Cre/lox system. Targeted pancreatic activation of the transgene was accomplished by injection of adenovirus carrying Cre into the pancreatic ducts and acini [152, 153]. Tumors in the model exhibit similarities to the human pancreatic ductal adenocarcinoma. Hras250 is deposited to National BioResource Project (NBRP Rat No.0568), and available [143].

Transgenic rats as carcinogenic models promise our understanding of the behavior of cancer in vivo, and will be useful to explore new therapeutic approaches. For carcinogenicity studies, *ras*H2 mice and p53^+/−^ mice are utilized because of their high susceptibility for carcinogens [154]. Several transgenic rats in Table 2 exhibit high sensitivity to carcinogens and oncogenic events are easily initiated. However, their background data are still not enough and amassed research evidence may be needed for applying them to short-term carcinogenicity tests. In this decade, gene-targeting technology using rats might be about to enter a new period. Gene-targeting technology using zinc-finger nucleases (ZFNs) allowed generation of the first knock-out rat in 2009 [155, 156]. And, generation of knock-out rats was achieved using rat ES cell-based technology in 2010 [157]. More recently, transcription activator-like effector nucleases (TALEN) and CRISPR/Cas9 systems were introduced to generate knock-out and knock-in rats [158]. The study utilizing gene-modified animals might be stepped up by advent of knock-out rats. p53 knock out rats are expected to be highly susceptible to chemical carcinogens. They will be applied to short-term carcinogenicity assays even though the p53 knock out rats and p53 knock out mice reveal differing phenotypes [159]. Recently, the data with transgenic rats for evaluation of carcinogenic potency of chemicals have been remarkably accumulated. Transgenic rats for mutagenesis and carcinogenesis will be principal models in future carcinogenesis studies and drug developments.

## Perspective

Development of transgenic rats for mutagenesis opened a possibility to use them in repeat dose toxicity assays, thereby enabling general toxicity and genotoxicity assays in same rats [7, 160]. This approach is consistent with the principle of 3Rs (Replacement, Refinement and Reduction) of animal use in laboratory experiments. For this purpose, SD and F344 *gpt* delta rats were compared with non-transgenic SD and F344 rats for their toxic and genotoxic responses to diethylnitrosamine (DEN) and di(2-ethylhexyl)phthalate (DEHP) [161]. DEN induced similar levels of GST-P foci in the liver of both transgenic and non-transgenic rats. DEN but not DEHP increased *gpt* and Spi^−^ mutation frequency in the liver of transgenic rats. It was concluded that SD and F344 *gpt* delta rats exhibited comparable toxic and genotoxic responses to DEHP and DEN to those with non-transgenic SD and F344 rats. Therefore, introduction of transgenic rats to repeat dose toxicity assays seems a promising future of toxicology and genotoxicology studies. However, standardization of assay procedures still needs more experimental results and discussion. For example, 4 weeks treatment of chemicals is recommended for gene mutation assays with transgenic rats by OECD TG488. However, KBrO_3_ at 500 ppm in drinking water needs 9 weeks to detect Spi^−^ mutations in the kidney of rats although 8-oxoguanien in DNA is formed by 1 week treatment [119]. Amosite at 2 mg by intratracheal instillation induced *lacI* mutations in the lung after treatment period of 16 weeks but not after 1 week administration [126]. Administration periods longer than 4 weeks may be required to detect mutations induced by weak mutagens or oxidative stress such as inflammation.

Epigenetic influence of environmental chemicals is an important research area in a field of chemical carcinogenesis. It is well documented that methylation of cytosine and demethylation of 5-MC in DNA, and methylation, acetylation and phosphorylation of histone strongly affect the expression of genes and the phenotypes [162, 163]. Perhaps epigenetic changes may underlie the mechanisms of some of non-genotoxic carcinogens. In fact, one of the mechanisms of nickel-induced carcinogenesis is epigenetic alterations [164]. Although there is no literature where Big Blue rats or *gpt* delta rats are used for epigenetic studies as far as we searched, one paper reported mechanical irritation increased mutation frequency in bladder without alteration of mutation spectrum [25]. It may be interesting to investigate the epigenetic alterations associated with chemical treatments when the mutation frequency increases without changing the mutation spectrum. Perhaps methylation status of cytosine in DNA may be altered by the treatments.

Recent advance in genome editing technology such as CRISPR/Cas9 has an impact on biomedical research including mutagenesis and carcinogenesis. In the near future, knock-out and knock-in rats will be generated more extensively. Aflatoxin B_1_ and tamoxifen induce tumors in rats more frequently compared with mice [18, 58]. Thus, genetic factors that affect the carcinogenesis may be investigated with knock-out or knock-in rats. In addition to the genome editing technology, DNA sequence analysis with NGS is greatly evolved recent years. NGS has been employed to characterize *lacZ* mutations in transgenic mice for mutagenesis [165] and for exome analysis of ENU-induced germ line mutation in *gpt* delta mice [166]. DNA adducts and mutation signature in human cancer may reflect the history of exposure of the patients to environmental chemicals. Since sensitivity of mass spectrometer has been increased substantially, relationships among DNA adducts, mutations and human cancer will be more extensively studied.

## Conclusions

Although mutation is an underlying mechanism of carcinogenesis, the literature reviewed here exhibits complex relationships between in vivo mutagenesis and carcinogenesis even for genotoxic carcinogens. The simplest relationship between mutagenesis and carcinogenesis is that mutations are induced only in the target organs or sub-organs for carcinogenesis. However, mutations are induced by PhIP not only in target lobe of prostate but also in non-target lobes [41]. PhIP induces mutations in the colon of male and female rats while it induces tumors predominantly in male rats. Phenacetin induced mutations in the liver (a non-target organ) much more strongly than in the kidney (the target organ) [52]. Similarly, TDBP induces mutations in the cortex of kidney (a non-target site) more extensively than outer medulla of kidney (the target site) [47]. These results suggest that the highest mutation induction does not coincide with the localization of tumors. The relationship between DNA adduct and mutation is not simple too. Leucomalachite green induces DNA adducts in the liver of rats but no mutations are induced [79]. Bitumen fumes induces DNA adducts in the lung without induction of detectable mutations [132]. Obviously, factors other than mutation such as cell proliferation strongly affect the carcinogenesis. Nevertheless, transgenic rat models for mutagenesis and carcinogenesis are useful tools for various purposes such as regulation of chemicals, chemoprevention studies and mechanistic investigations. Mutation spectra induced by chemical exposure with transgenic rats may be useful to interpret the mutation signatures of human cancer. Advanced sequencing technology coupled with transgenic rat models may contribute significantly to further development of research on chemical mutagenesis and carcinogenesis.

## Abbreviations

2,4-DAT: 2,4-diaminotoluene
2,6-DAT: 2,6-diaminotoluene
3-MCPD: 3-monochloropropane-1,2-diol
3Rs: Replacement, Refinement and Reduction
4-OH-PCB3: 4-hydroxy-PCB3
5-BT: 5-*p*-dimethylaminophenylazaobenzthiazole
5-MC: 5-methylcytosine
6-BT: 6-*p*-dimethylaminophenylazaobenzthiazole
BP: Benzo[*a*]pyrene
CPA: Cyproterone acetate
Cx32: Connexin 32
Cx32Δ transgenic: *Connexin 32* dominant-negative transgenic rats
DAS: Diallyl sulfide
DE: Diesel exhaust
DEHP: Di(2-ethylhexyl)phthalate
DEN: Diethylnitrosamine
DMBA: 7, 12-dimethylbenz[a]anthracene
DMH: Dimethyl hydrazine
DMN: Dimethyl nitrosamine
*E. coli*: *Escherichia coli*
ENU: *N*-ethyl-*N*-nitrosourea
F344: Fischer 344
GST-P: Glutathione S-transferase placental form
Hras128: Human c-Ha-*ras* proto-oncogene transgenic rats
IQ: 2-amino-3-methylimidazo[4,5-*f*]quinoline
KBrO_3_: Potassium bromate
MeIQx: 2-amino-3,8-dimethylimidazo[4,5-*f*]quinoxaline
MNU: *N*-methyl-*N*-nitrosourea
NGS: Next-generation DNA sequencer
Ni_3_S_2_: Nickel subsulfide
NMBA: *N*-nitrosomethylbenzylamine
OECD: Organization for Economic Co-operation and Development
PCB3: 4-monochlorobiphenyl
PhIP: 2-amino-1-methyl-6-phenylimidazo[4,5-*b*]pyridine
PoD: Points of Departure
SD: Sprague—Dawley
SV40: Simian virus 40
TALEN: Transcription activator-like effector nucleases
TCDD: 2,3,7,8-tetrachlorodizenzo-*p*-dioxin
TDBP: Tris(2,3-dibromopropyl)phosphate
TRAP: Probasin-SV40 T antigen transgenic rats
WHO: World Health Organization
ZFNz: Zinc-finger nucleases
